# Dietary change influences the composition of the fecal microbiota in two rescued wild raccoon dogs (*Nyctereutes procyonoides*)

**DOI:** 10.3389/fmicb.2024.1335017

**Published:** 2024-02-09

**Authors:** Hailong Li, Lei Bao, Tianming Wang, Yu Guan

**Affiliations:** ^1^National Forestry and Grassland Administration Key Laboratory for Conservation Ecology in the Northeast Tiger and Leopard National Park, Beijing Normal University, Beijing, China; ^2^College of Geography and Ocean Science, Yanbian University, Yanji, China; ^3^Department of Agriculture, Forestry and Bioresources, College of Agriculture and Life Science, Seoul National University, Seoul, Republic of Korea

**Keywords:** gut microbiota, raccoon dogs (*Nyctereutes procyonoides*), wildlife rescue, 16S rRNA gene, high-throughput sequencing

## Abstract

The gut microbiota of wild animals, influenced by various factors including diet, nutrition, gender, and age, plays a critical role in their health and disease status. This study focuses on raccoon dogs (*Nyctereutes procyonoides*), a commonly found wild animal, and its gut microbiota composition in response to dietary shifts. The study aimed to compare the fecal bacterial communities and diversity of rescued raccoon dogs fed three different diet types (fish and amphibians, mixed protein with maize, and solely maize) using high-throughput sequencing. Results indicated that the dietary composition significantly influenced the gut microbiota, with notable differences in the abundance of several key phyla and genera. The study identified *Firmicutes* as the dominant phylum in all diet groups, with notable variations in the relative abundances of *Bacteroidota*, *Proteobacteria*, and *Verrucomicrobiota*. Notably, the group solely fed maize exhibited a significant increase in *Proteobacteria*, potentially linked to dietary fiber and lignin degradation. The genus-level analysis highlighted significant differences, with *Lactobacillus* and *Bifidobacterium* responding to dietary shifts. The genus *Akkermansia* in *Verrucomicrobiota* can be identified as a marker for assessing the health of the gut and deserves further investigation. Gender-specific differences in the gut microbiota were observed, highlighting the influence of individual variation. Furthermore, the analysis of bacterial functions suggested a connection between diet and host metabolism, emphasizing the need for further research to understand the complex mechanisms underlying the relationship between dietary composition and gut microbiota in wild animals. These findings provide crucial insights into conservation and rescue efforts for wild animals.

## Introduction

The gut microbiota is a complex ecosystem that encompasses trillions of bacteria, which could affect the health and disease of wild animal hosts through many influencing factors, such as diet, nutrition, gender, and age (Flint et al., 2012; Scott et al., 2013; Fransen et al., 2017). While major bacterial species may exhibit stability, the fundamental composition and structure of the gut microbiota can show significant variability in some instances (Leeming et al., 2019). Diet is considered an important influencing factor that makes a contribution to interpreting bacterial structural variations in both mice and humans (Zhang et al., 2010; Rothschild et al., 2018). The bacterial composition can respond rapidly to the dietary intervention within 24–48 h (Sonnenburg and Bäckhed, 2016). Moreover, short- and long-term dietary interventions have been demonstrated to alter the bacterial diversity of the gut microbiota. Wild animals generally face a variety of challenges and threats, such as habitat fragmentation and seasonal food scarcity. Thus, research and comprehension of the variation in gut microbiota may provide advanced and non-invasive methods for wild animal conservation and rescue (Waite and Taylor, 2015; Wu et al., 2021).

Raccoon dogs (*Nyctereutes procyonoides*) are a common animal belonging to the Canidae family in the order Carnivora, which was originally distributed in East Asia, including Japan, northern Vietnam, Korea, and Siberia. Then the population of raccoon dogs expanded widely from Western Russia to many countries in Europe due to their value in the fur trade and hunting (Sutor et al., 2014; Chueca et al., 2021). At present, China is one of the countries with the largest number of raccoon dogs breeding in the world (Xu et al., 2016; Qin et al., 2019). Wild raccoon dogs are listed as a state second-class protected animal in China, while it is listed as the least concern by the International Union for Conservation of Nature (IUCN) due to the stable population trend. The raccoon dogs are typical omnivores that generally feed on amphibians, fish, insects, invertebrates, birds, rodents, and various plants (Kauhala et al., 1993; Sutor et al., 2010), of which the diet composition shift was observed seasonally. In addition, when encountering severe winter and food scarcity, the raccoon dogs would choose to hibernate to decrease their metabolism as much as possible.

A large number of researches have covered various fields due to the stable population of raccoon dogs and the need for fur farming, such as distribution (Helle and Kauhala, 1991; Kauhala and Helle, 1995), diet (Sutor et al., 2010; Drygala et al., 2013), behaviors (Saeki et al., 2007; Rudert et al., 2011), and genetic diversity (Pitra et al., 2010; Zhang and Chen, 2010; Drygala et al., 2016). Although several studies on the gut microbiota of raccoon dogs were reported (Peng et al., 2018, 2019; Liu et al., 2020), the experimental resources were limited to captive individuals. However, the influence on the gut microbiota due to the alteration of dietary composition in wild-rescued individuals has yet to be studied.

The present study aimed to more explicitly delineate the novel contributions relative to the existing literature by comparing the fecal bacterial communities and diversity of wild-rescued raccoon dogs from three diet types, employing the Illumina MiSeq platform for sequencing the V3–V4 region of the 16S rRNA gene. The results are anticipated to furnish essential data for a deeper understanding of the fecal microbiota in wild raccoon dogs with three different diet compositions and offer insights into the rescue of wild animals.

## Materials and methods

### Ethics statement

This study was approved and performed in accordance with permission from the Ethics and Animal Welfare Committee of Beijing Normal University (approval reference number: CLS-EAW-2020-037).

### Sample collection

A total of 27 fecal samples were collected from 2 raccoon dogs (1 male and 1 female), which were rescued in Northeast China Tiger and Leopard National Park during December 2020 and March 2021. Raccoon dogs were then raised in special cages outdoor, in which the real-time monitoring equipment was installed. Three diets were designed according to the general feeding habits of wild raccoon dogs (A: fish 68.00%, Dybowski’s frog 25%, and *Cambaroides dauricus* 7%; B: fish 29%, Dybowski’s frog 14%, *Cambaroides dauricus* 14%, and maize 43%; C: maize 100%). Each diet was fed for more than 2 weeks. Fecal sample collections were conducted during the middle of each feeding period, and samples were also grouped by the three different diets (group A: 10 samples, group B: 10 samples, and group C: 7 samples, respectively). Two wild raccoon dogs wearing GPS monitoring collars were released to the site where they were originally rescued.

All the fecal samples were transported into the laboratory and stored at −80°C for further study.

### DNA extraction and Illumina MiSeq sequencing

The total genomic DNA from fecal samples was extracted using the QIAamp Stool Mini Kit (51504) (Qiagen, Germany) according to the manufacturer’s instructions. The quality of the extracted DNA was assessed by NanoDrop 2000. The V3–V4 region of the bacterial 16S rRNA gene was amplified using the primers 338F (5′-barcode-ACTCCTACGGGAGGCAGCAG-3′) and 806R (5’-GGACTACHVGGGTWTCTAAT-3′). Amplification was carried out in 20-μL reactions: 4 μL of 5× FastPfu Buffer, 2 μL of 2.5 mM dNTPs, 0.8 μL of each primer (5 mM), 0.4 μL of FastPfu Polymerase, and 10 ng of template DNA. The PCR cycle comprised an initial denaturation at 95°C for 3 min, followed by 25 cycles of 95°C for 30 s, 55°C for 30s, 72°C 30s, and a final extension at 72°C for 5 min.

After being purified and quantified using the AxyPrep DNA Gel Extraction Kit (AP-GX-500) (Axygen Biosciences, Union City, CA, United States) and QuantiFluorTM-ST (Promega, United States), amplicons were sequenced on an Illumina MiSeq platform.

### Sequences processing and data analysis

Raw files were demultiplexed and quality-filtered using QIIME (version 1.9.1). The sequences were clustered into operational taxonomic units (OTUs) at 97% similarity using UPARSE (version 7.1). The taxonomy of 16S rRNA sequences was analyzed using the RDP Classifier^1^ against the Silva (SSU 138) 16S rRNA database with a confidence threshold of 70%.

Alpha diversity indices were calculated by mothur (version 1.30.2). Then a Wilcoxon rank-sum test was performed to test whether there was a significant difference in alpha diversity indices among groups. The rarefaction curves, the histogram of relative abundance for species, and the community heatmap were calculated and depicted by R (version 3.3.1). For the analysis of beta diversity, the unweighted UniFrac distance matrixes were generated using QIIME (version 1.9.1). The hierarchical clustering trees, principal co-ordinate analysis (PCoA), and non-metric multi-dimensional scaling analysis (NMDS) were performed with R (version 3.3.1). The Kruskal–Wallis H-test was used to evaluate the significance level of species abundance differences and obtain species with significant differences between groups. Finally, bacterial functions were predicted by PICRUSt based on the high-quality sequences we obtained.

The data were analyzed on the free online platform of the Majorbio Cloud Platform.^2^

The data for our study are available in the NCBI Sequence Read Archive (SRA): PRJNA1036717.

## Results

### Overall sequencing data

Across the 27 fecal samples of 2 rescued wild raccoon dogs, a total of 1,144,436 clean reads with an average length of 415 bp were obtained after quality control by high-throughput sequencing on the Illumina MiSeq platform. The total number of clean reads, base pairs, and the mean length of reads in all samples are shown in Supplementary Table S1. Then the clean reads were classified into 326 OTUs with a 97% similarity threshold in 27 fecal samples, and a total of 10 phyla, 17 classes, 42 orders, 70 families, and 150 genera were detected among the fecal microbiota of the raccoon dogs. The alpha diversity indices, including Sobs, Shannon, Chao 1, Ace, and Good’s coverage, are shown in Table 1.

**Table 1:** Alpha diversity of gut microbiota from raccoon dogs.

Sample	Sobs	Shannon	Simpson	Ace	Chao	Good’s coverage
A1	163	3.462	0.059	179.576	186.333	0.999
A2	183	3.564	0.052	207.426	205.556	0.999
A3	178	3.687	0.040	193.071	193.400	0.999
A4	185	3.399	0.069	200.426	197.833	0.999
A5	193	3.480	0.058	218.824	222.176	0.999
A6	167	3.293	0.060	181.789	182.333	0.999
A7	123	3.061	0.072	136.001	137.250	0.999
A8	181	3.173	0.102	197.683	204.077	0.999
A9	188	3.644	0.046	210.546	209.938	0.999
A10	156	3.042	0.087	181.346	185.077	0.999
B1	94	2.302	0.149	118.913	115.083	0.999
B2	101	2.214	0.163	158.654	132.500	0.999
B3	71	1.948	0.204	81.391	77.600	1.000
B4	57	1.863	0.305	69.286	66.750	1.000
B5	46	1.555	0.367	51.966	55.333	1.000
B6	165	3.147	0.079	185.563	185.647	0.999
B7	180	3.451	0.061	205.018	222.273	0.999
B8	174	3.516	0.050	202.727	209.769	0.999
B9	163	3.232	0.079	180.569	178.813	0.999
B10	155	2.847	0.133	164.084	163.000	0.999
C1	98	1.797	0.428	125.338	174.500	0.999
C2	89	1.177	0.615	103.441	104.545	0.999
C3	111	2.342	0.189	116.842	113.571	1.000
C4	134	2.874	0.140	147.074	149.545	0.999
C5	131	2.507	0.208	147.557	145.615	0.999
C6	138	1.959	0.329	165.039	163.375	0.999
C7	179	3.494	0.060	199.936	213.364	0.999

The rarefaction curves were calculated and are depicted in Supplementary Figure S1, which indicated that the sample size we used was large enough to characterize the basic composition of the fecal microbiota of raccoon dogs.

### Microbiota composition and relative abundance

**Figure 1:**
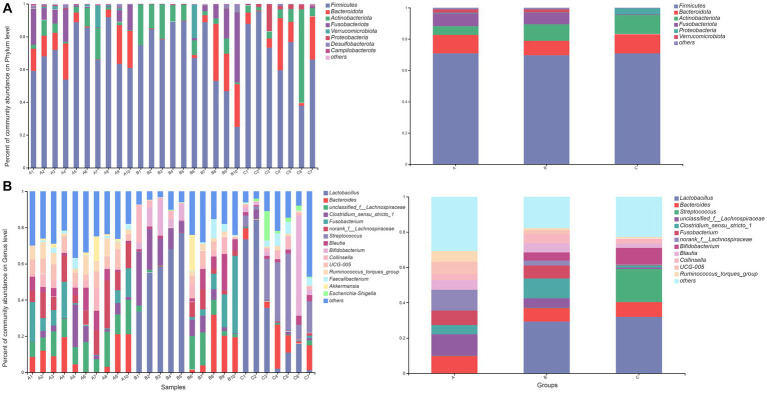
Bar plot of relative abundance in the gut microbiota of raccoon dogs at the phylum **(A)** and genus **(B)** levels. The horizontal axis represents the sample names, while the vertical axis represents the proportion of species in each sample. Different colored bars represent different species, and the length of the bars represents the relative proportion of each species.

At the phylum level, the fecal microbiota of raccoon dogs from the A group was dominated by *Firmicutes* (70.97%), *Bacteroidota* (11.66%), and *Fusobacteriota* (8.25%), which accounted for over 90.00% of sequences in the A group. The fecal microbiota from the B group was dominated by *Firmicutes* (69.80%), *Actinobacteriota* (10.50%), and *Bacteroidota* (9.08%). For the C group, *Firmicutes* (70.96%) was the most predominant phylum, followed by *Actinobacteriota* (12.23%) and *Bacteroidota* (3.90%). The bacterial compositions at the phylum (A) and genus (B) levels in the three groups are demonstrated in Figure 1.

**Figure 2:**
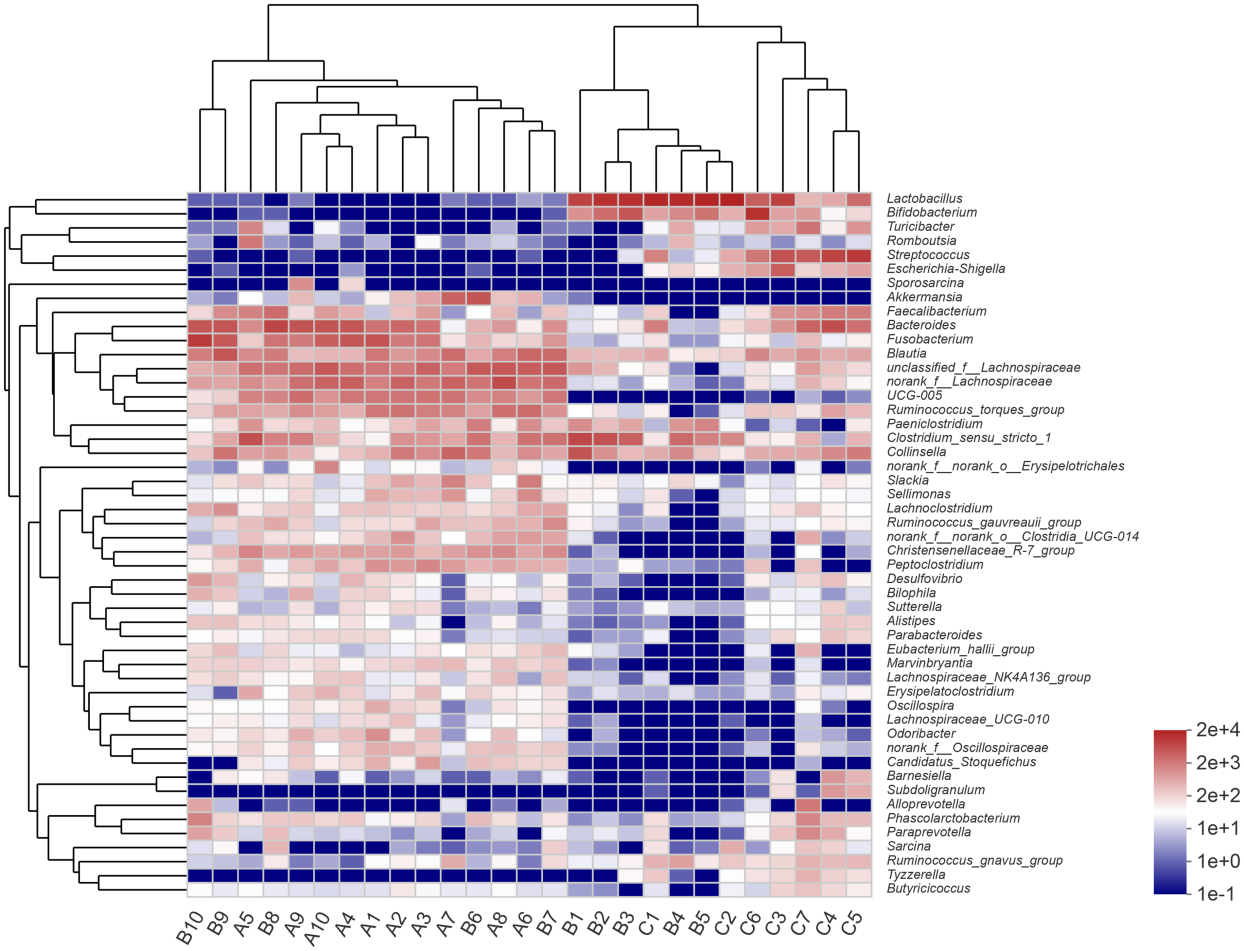
Heatmap of clustering for species abundance. The horizontal axis represents the sample names, while the vertical axis represents the species names. The abundance variation of different species in each sample is displayed using color gradients. The numerical values corresponding to the color gradient are shown on the right side of the graph.

At the genus level, the most abundant genera in the A group were *unclassified_f__Lachnospiraceae* (12.16%), *norank_f__Lachnospiraceae* (11.90%), and *Bacteroides* (9.87%). The most abundant genera in the B group were *Lactobacillus* (29.29%), *Clostridium_sensu_stricto_1* (11.11%), and *Bacteroides* (7.71%). *Lactobacillus* (31.96%), *Streptococcus* (18.72%), and *Bifidobacterium* (9.27%) were the most dominant genera in the C group. The result of community heatmap analysis at the genus level is depicted in Figure 2.

**Figure 3:**
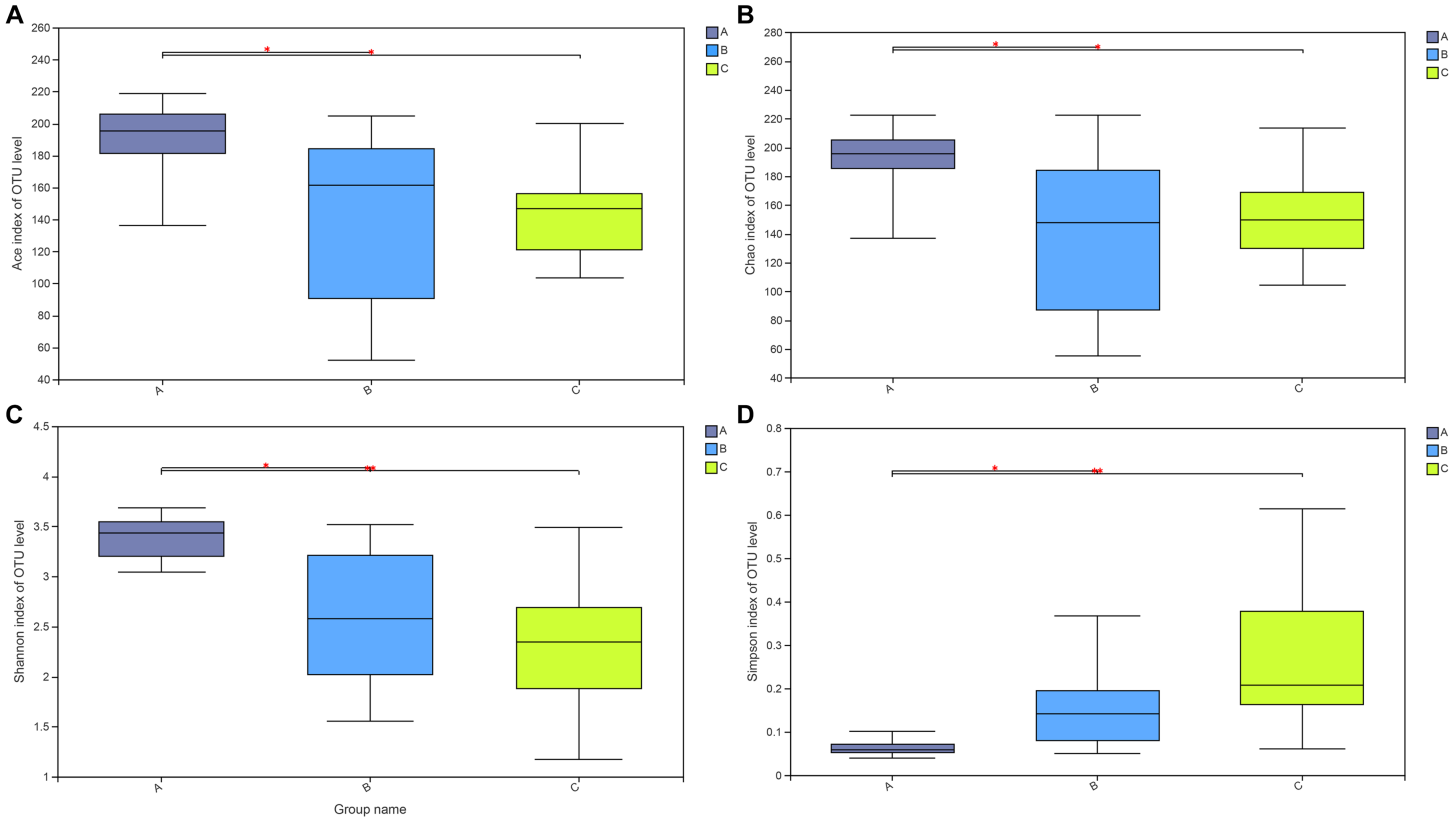
Wilcoxon rank-sum test for alpha diversity indices of raccoon dog samples, including Ace **(A)**, Chao **(B)**, Shannon **(C),** and Simpson **(D)** indices. This graph displays the significant differences among the three groups of samples. The horizontal axis represents the group names, while the vertical axis represents the range of indices for each group.

Additionally, the Wilcoxon rank-sum test was applied to determine if the alpha diversity indices were significantly different among rescued wild raccoon dogs with different diets. The test results (Figure 3) among the three groups showed that the alpha diversity indices (Ace (A), Chao (B), Shannon (C), and Simpson (D) index) were all significantly higher in the A group than those in the B and C groups (*p* < 0.05), which indicated that the community richness and diversity of fecal microbiota were greater in the A group.

### Beta diversity and community structure

**Figure 4:**
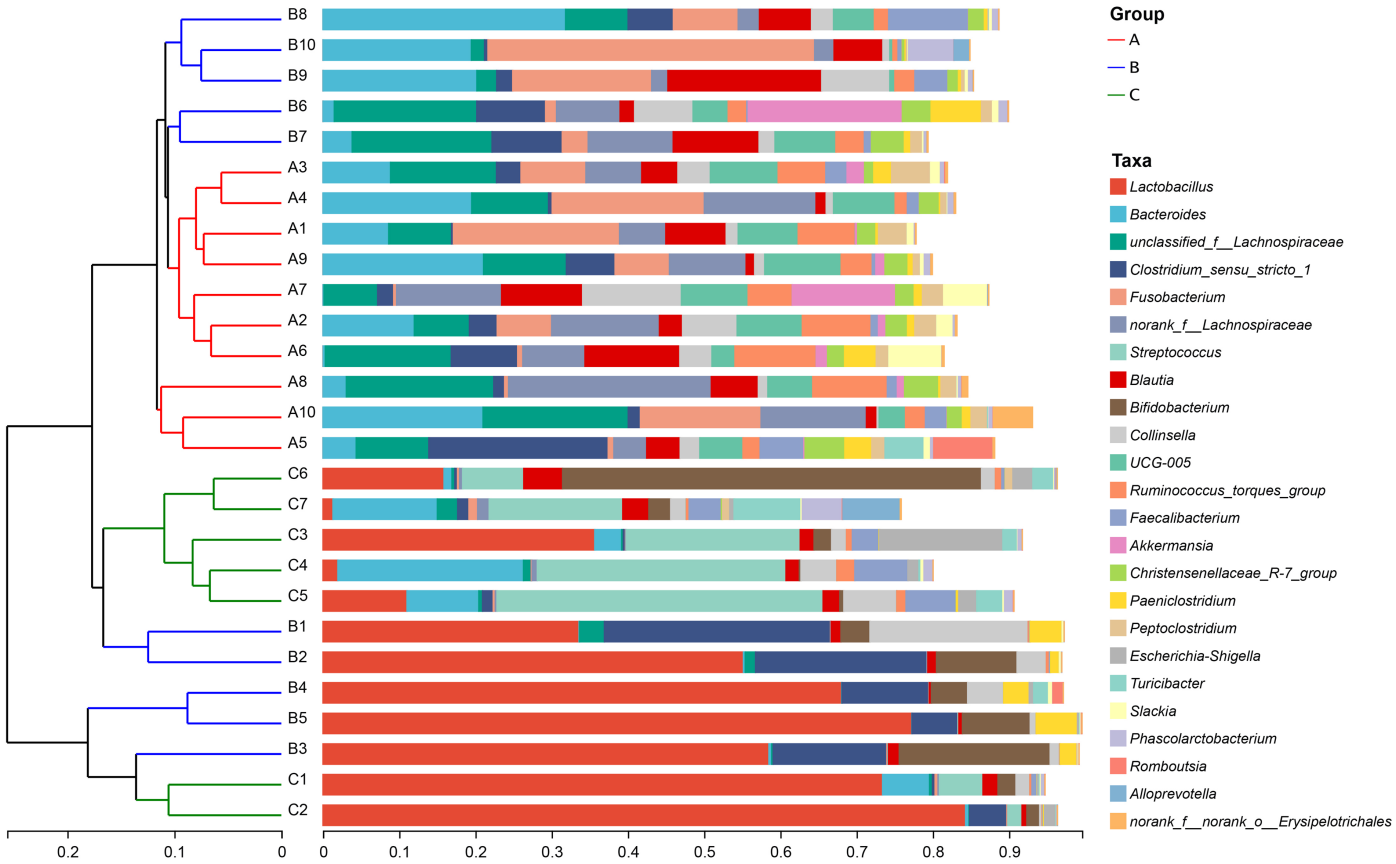
Hierarchical clustering tree. The tree was generated based on the unweighted distance matrix at the genus level.

**Figure 5:**
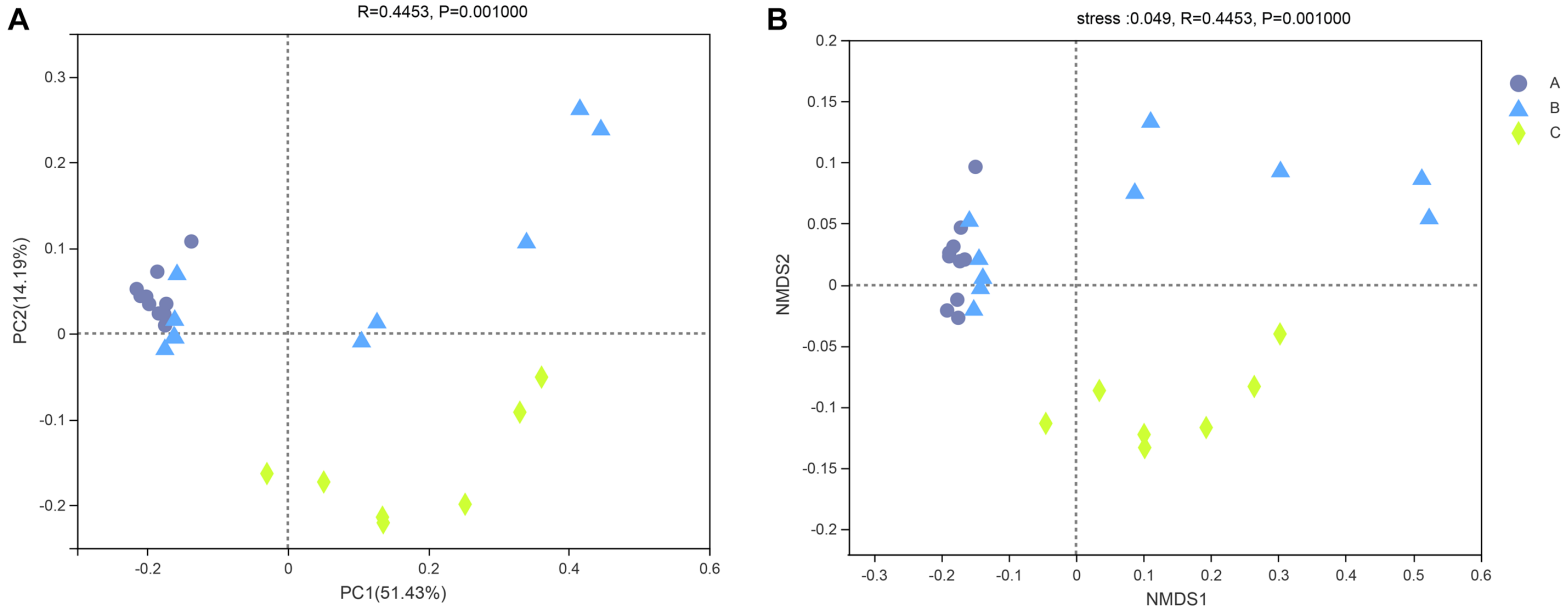
PCoA **(A)** and NMDS **(B)** of the intestinal bacterial community structures of the raccoon dogs. Different colors or shapes of points represent samples from different groups. The closer the points of two samples are, the more similar their species composition is.

The similarity of the bacterial community structure of fecal microbiota from three breeding conditions was shown by the hierarchical clustering trees (Figure 4), which were generated by UPGMA (unweighted pair-group method with arithmetic mean) with the unweighted UniFrac distance matrixes. The dendrogram indicated that samples from the A (A1–A10) and B (B6–B10) groups clustered together, while the other samples from the B (B1–B5) group located on similar branches with those from the C group (C1–C7). Notably, samples marked as B1–B5 and B6–B10 came from male and female raccoon dogs, respectively. The obvious different branches we observed after the dietary change may be due to the gender difference of raccoon dogs. Furthermore, to assess the discrepancy among the three groups, we generated the principal co-ordinates analysis (PCoA) plot (Figure 5A) and the non-metric multi-dimensional scaling (NMDS) plot (Figure 5B) with the unweighted UniFrac distance matrixes. The results of PCoA and NMDS were also similar to those in the hierarchical clustering trees, which showed that samples from the A and C groups tended to gather together within groups, while samples from male and female raccoon dogs in the C group tended to cluster separately from each other.

**Figure 6:**
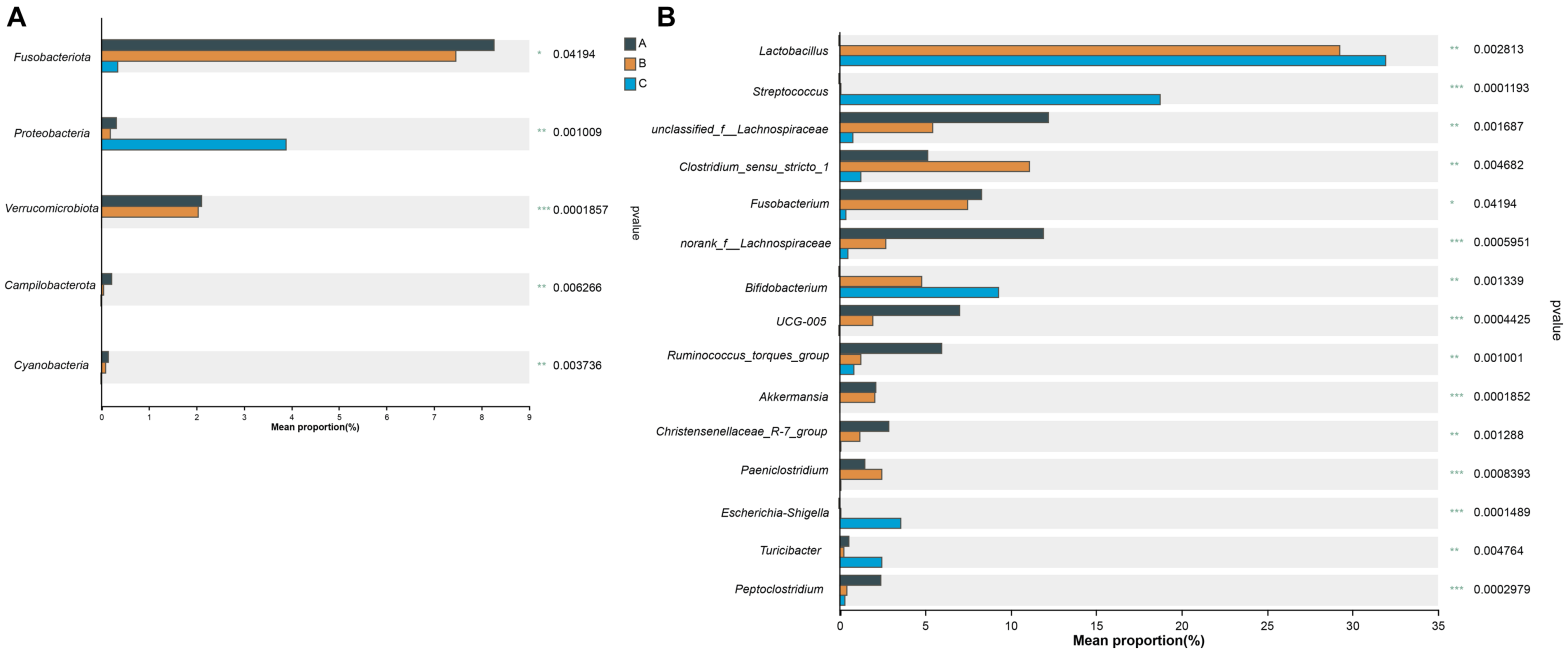
Kruskal–Wallis H-test bar plot at the phylum **(A)** and genus **(B)** level. The X-axis represents different groups, with boxes of different colors representing different groups. The Y-axis represents the average relative abundance of a certain species in different groups.

The core bacterial species in all 27 fecal samples were significantly different at the phylum (5 phyla) (Figure 6A) and genus (15 genera) (Figure 6B) levels, as demonstrated in the Kruskal–Wallis H-test bar plot.

**Figure 7:**
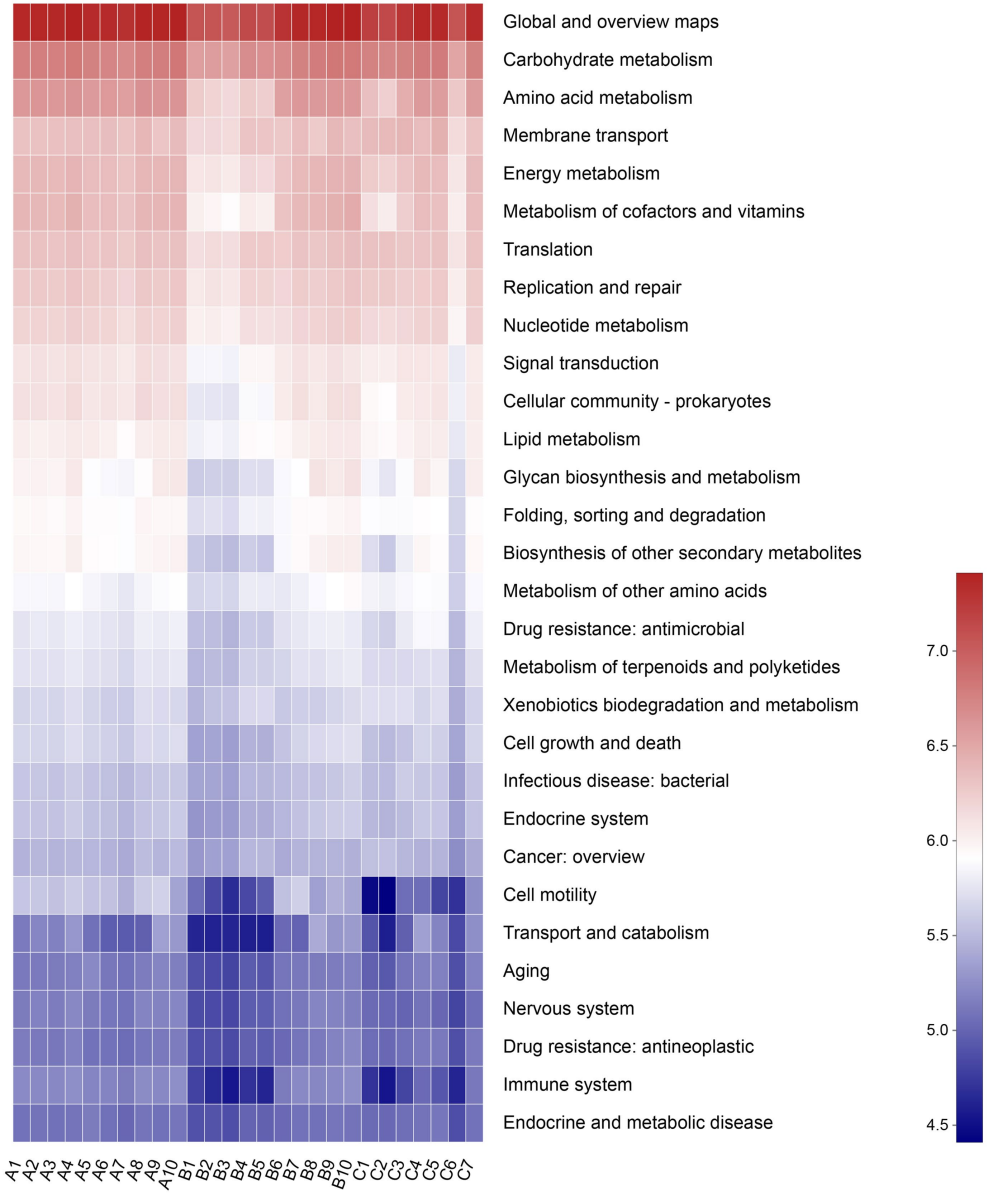
Abscissa represents the sample name, and the ordinate represents the pathway level 2 functional names. The sample’s different functional abundance variations are displayed using a color gradient for the color blocks. The legend represents the numerical values represented by the color gradient.

A total of 5,709 Kos (KEGG orthologs) and 46 KEGG pathways were mapped based on the high-throughput sequencing. The secondary KEGG pathways, such as metabolism and genetic information processing, were detected to be related to the gut microbiota of raccoon dogs (Figure 7).

## Discussion

This study characterized 27 fecal samples of raccoon dogs with three different diets by sequencing the V3–V4 region of the 16S rRNA gene using the Illumina MiSeq platform. Then we analyzed the bacterial composition in the gut microbiota and characterized the differences among samples. Raccoon dogs are a common canine species that has been studied in many fields, including the effects of the rapid population dispersals and the gut microbiota of breeding individuals, as well as the risks for humans caused by the occurrence of their diseases and parasites (Shibata and Kawamichi, 1999; Mulder, 2013; Liu et al., 2020; Kjær et al., 2021). However, the influences for the gut microbiota caused by the dietary composition shift for raccoon dogs were currently unknown, especially for the wild individuals. Therefore, to figure out the relationships between different diets and bacterial communities of the fecal microbiota from wild-rescued raccoon dogs, high-throughput sequencing technology was applied.

### Microbiota composition and relative abundance

The characterization of microbial communities in the fecal samples of wild raccoon dogs showed that the basic composition and structure of the gut microbiota were significantly different among the three groups with different diets. *Firmicutes* was the most predominant phylum in all three groups, which was consistent with the previous studies on gut microbiota for canines, such as farmed raccoon dogs (Liu et al., 2020), wolves (*Canis lupus*) (Zhang and Chen, 2010), red foxes (*Vulpes vulpes*), corsac foxes (*Vulpes corsac*) (Wang et al., 2022), and domestic dogs (*Canis lupus familiaris*) (Deng and Swanson, 2015). The third dominant phylum in the A group was *Fusobacteriota*, which was significantly higher (*p* = 0.042) than the other groups. For the B group and C group, *Bacteroidota* was another most abundant phylum that ranked after *Firmicutes* and *Actinobacteriota*.

Additionally, although *Proteobacteria* was the fourth dominant phylum in the C group, the relative abundance was significantly higher (*p* = 0.001) than in the A group and B group. *Proteobacteria* is the largest bacterial phylum and includes many pathogenic bacteria that could cause diseases, such as diarrhea, inflammation, and inflammatory bowel disease (Hermes et al., 2009; Carvalho et al., 2012). Members of *Proteobacteria* have been reported to be closely associated with the degradation of lignin from bamboo, which is a major food resource and beneficial for the giant panda (Fang et al., 2012). Coincidentally, the diet of raccoon dogs in the C group was only comprised of maize. Furthermore, *Proteobacteria* are hypothesized to contribute to the homeostasis of the anaerobic environment of the gastrointestinal tract (Moon et al., 2018). Similarly, an increase in the relative abundance of *Proteobacteria* was also observed when potato fiber was supplemented into food as a prebiotic for domestic dogs (Panasevich et al., 2015). In our study, the significantly higher relative abundance of *Proteobacteria* in the C group may be caused by the pure vegetarian diet, which could promote the degradation process of fiber and lignin in food more efficiently. Samples from more different individuals should be collected to avoid the influences caused by individual differences.

Another phylum with significantly higher relative abundance (*p* < 0.001) was *Verrucomicrobiota*. The relative abundance of this *Verrucomicrobiota* in both the A group (2.09%) and the B group (2.03%) was higher than in the C group (0.00%). *Verrucomicrobiota* exists mainly in aquatic and soil environments (Op den Camp et al., 2009; Bergmann et al., 2011). *Akkermansia* in *Verrucomicrobiota* was known as “lean bacteria” (Belzer and de Vos, 2012). Studies have shown that the abundance of *Verrucomicrobiota* showed a significant decrease resulting from high-fat diet consumption (Wang et al., 2019). The diets of the A group and B group were mainly composed of fish, frogs, and crayfish, which contain a lot of protein. Therefore, contrary to the results of the microbial community shift due to the high-fat diet, our study indicated that the high-protein diet for wild raccoon dogs led to a significant increase in *Verrucomicrobiota* in the fecal microbiota.

Moreover, the mucus-degrading bacterium *Akkermansia muciniphila* in *Verrucomicrobiota* was considered a hallmark of a healthy gut for its kinds of abilities, such as anti-inflammatory and immunostimulant properties (Fujio-Vejar et al., 2017). The higher relative abundance of *Verrucomicrobiota* in A group and B group in the gut microbiota of raccoon dogs, as well as the genus *Akkermansia*, suggested that the diversity of diet composition was one of the most important influencing factors for gastrointestinal health.

At the genus level, *Lactobacillus* (31.96%) was the most abundant genus that showed a significant difference (*p* = 0.003) in the C group compared to the A group (0.004%), while *unclassified_f__Lachnospiraceae* (12.16%) was the most common genus in the A group. Previous studies have shown that *Lactobacillus* is beneficial for the host in many aspects, including immune modulation and alleviation of allergies (Konstantinov et al., 2008; Baldassarre et al., 2010). Novel members of *Lactobacillus* have also been found in the intestinal tracts of many animals, such as pigeons (Baele et al., 2003), horses (Morotomi et al., 2002), and pigs (Pedersen and Roos, 2004). The high β-xylosidase level has been reported in the presence of *Lactobacillus* in the gut microbiota, and β-xylosidase plays a crucial role in the complete degradation of xylan in the lignocellulose of hemicellulose (Ruggiero et al., 2009; Ye et al., 2017). In our study, the high relative abundance of *Lactobacillus* was first observed along with the supplement of maize in the diet in the B group (29.29%), indicating that the hemicellulose and cellulose in maize from dietary shift resulted in the increase of *Lactobacillus*.

*Bifidobacterium* was another genus with significantly higher (*p* = 0.001) abundance when the diet was comprised of maize. Similarly, *Bifidobacterium* is distributed in the gastrointestinal tract of various animals (Alberoni et al., 2019; Modesto et al., 2019). Carbohydrates are the main nutrients for the growth of *Bifidobacterium* and can modulate the colonization of this widespread genus. The increase in relative abundance of *Bifidobacterium* may also result from the supplementation of maize, which suggests that the digestion of lots of carbohydrates in raccoon dogs’ diet needs more *Bifidobacterium*. Thus, it is necessary to study the evolutionary relationship between *Bifidobacterium* and host animals in future research.

### Beta diversity analysis and community structures

To investigate the similarities or differences of bacterial communities in fecal samples, the unweighted UniFrac distance matrixes were calculated and then used in the generation of the hierarchical clustering trees, as well as the plots of PCoA and NMDS. In our study, the results of the three analyses above all showed that the fecal samples from raccoon dogs with the same diet tended to cluster together. The other finding that should be paid attention to was that the samples in the B group were clustered into two groups, respectively, based on gender (samples marked as B1–B5 and B2–B6).

PICRUSt was applied to analyze the bacterial functions of raccoon dogs. The results showed that the functions of the microbial community of raccoon dogs mainly include metabolism, genetic information processing, environmental information processing, and cellular processes, which would provide deeper insights into the relationship between diet type and the metabolism of the host. However, the limited wild samples and accessible individuals from raccoon dogs are still difficulties that should be solved to comprehend the specific functions and mechanisms of the bacterial communities in the gut microbiota.

The analysis of the gut microbiota of 27 fecal samples from two wild-rescued raccoon dogs demonstrated a similar composition and structure of bacterial communities with the previous results for raccoon dogs (Liu et al., 2020) at the phylum level. Furthermore, the comparisons of the relative abundance in the present study among three groups with different diets showed that there were significant differences in the abundance and structure of bacterial communities at both phylum and genus levels. More than that, when maize was introduced into the diet in the B group, different responses of the gut microbiota were detected between male and female raccoon dogs. Generally speaking, the gut microbiota of mammals is affected by many factors, such as diet, gender, geography, and host genetics (Turnbaugh et al., 2009; Zhang et al., 2010). The differences among the three groups were only dietary compositions. Studies have found that the more diverse the diets, the more diverse the microbiota, suggesting that the microbiota with higher diversity would better adapt to the various variations (Heiman and Greenway, 2016). However, a significant decrease in the diversity of gut bacterial communities was observed in raccoon dogs, which resulted from the increased dietary diversity. Therefore, the diet composition of raccoon dogs, which experienced a change from total high proteins to a mixture of 57% proteins and 43% maize, was not the only main influencing factor that affected the gut microbiota. Indeed, the gut microbiota of female raccoon dogs in the B group was similar to samples in the A group, indicating that gender also played an important role in manipulating gastrointestinal homeostasis. Given that the variation of the gut microbiota resulting from individual differences is a non-negligible factor, more samples from different individuals should be collected and analyzed to further prove the results we obtained. In addition, the phyla and genus with significant differences in the C group showed that the digestion ability of carbohydrates and cellulose in diets was affected by different bacterial communities. The specific function and molecular mechanism should be studied with the combination of multiple omics, which could provide a deeper comprehension of the conservation and wild animal rescue in the future.

## Conclusion

In this study, high-throughput sequencing assessed how dietary composition affects the gut microbiota of rescued raccoon dogs, revealing significant microbial differences among groups with varied diets. *Firmicutes* prevalence was consistent, while distinct variations such as higher *Fusobacteriota* and *Proteobacteria* were observed. *Verrucomicrobiota* exhibited contrasting responses to high-protein and high-carbohydrate diets, with implications for gut health, notably through *Akkermansia*.

The genus-level analysis highlighted significant differences, with *Lactobacillus* and *Bifidobacterium* responding to dietary shifts, indicating their vital role in nutrient digestion. Diversity analysis revealed sample clustering based on similar diets and gender-based variations within one diet group. While diet was a prominent factor influencing the gut microbiota, individual differences and gender also contributed to microbial variations.

Further research is needed to uncover specific functions and molecular mechanisms. Collecting more samples and using multi-omics approaches will deepen our understanding of the intricate relationship between dietary composition, gut microbiota, and wildlife health. These insights are crucial for guiding conservation and rescue efforts for raccoon dogs and other wildlife in the future.

## Data availability statement

The datasets presented in this study can be found in online repositories. The names of the repository/repositories and accession number(s) can be found in the article/Supplementary material.

## Ethics statement

The animal study was approved by Ethic and Animal Welfare Committee, College of Life Science, Beijing Normal University. The study was conducted in accordance with the local legislation and institutional requirements.

## Author contributions

HL: Data curation, Formal analysis, Investigation, Methodology, Project administration, Resources, Software, Visualization, Writing – original draft, Writing – review & editing. LB: Conceptualization, Investigation, Methodology, Project administration, Resources, Supervision, Validation, Visualization, Writing – review & editing. TW: Conceptualization, Funding acquisition, Investigation, Project administration, Resources, Supervision, Writing – review & editing. YG: Conceptualization, Formal analysis, Funding acquisition, Methodology, Project administration, Resources, Software, Supervision, Visualization, Writing – original draft, Writing – review & editing.

## Footnotes

^1^ http://rdp.cme.msu.edu/

^2^ www.majorbio.com
