# Supplementary figures and images for: Dietary change influences the composition of the fecal microbiota in two rescued wild raccoon dogs (Nyctereutes procyonoides)
Source: Front Microbiol. 2024 Feb 9;15:1335017. doi: 10.3389/fmicb.2024.1335017 (PMC10884114; doi:10.3389/fmicb.2024.1335017)

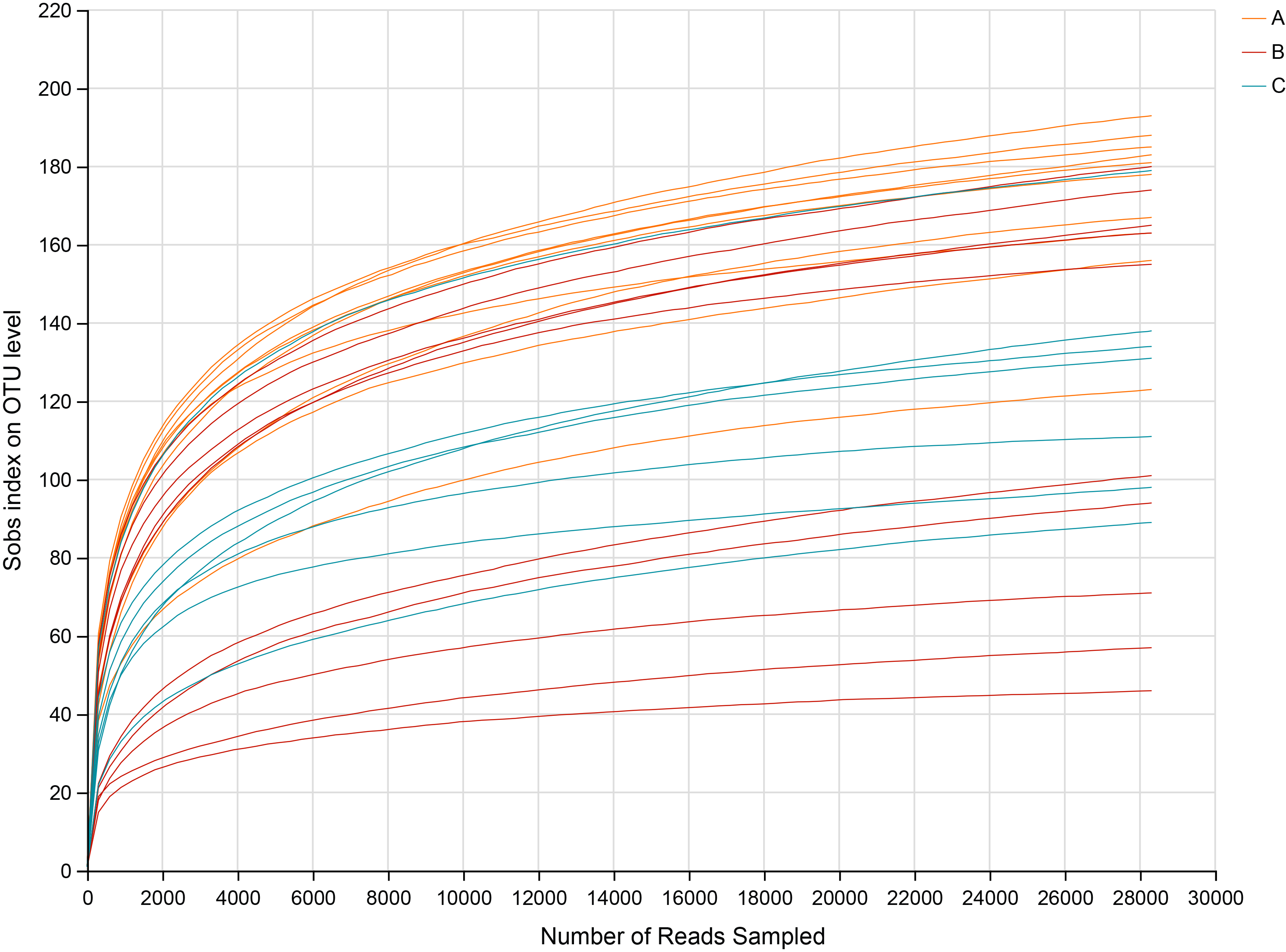

Supplement: Supplementary file 1 [file Image_1.TIF]
