# Supplementary material for: Dietary change influences the composition of the fecal microbiota in two rescued wild raccoon dogs (Nyctereutes procyonoides)
Source: Front Microbiol. 2024 Feb 9;15:1335017. doi: 10.3389/fmicb.2024.1335017 (PMC10884114; doi:10.3389/fmicb.2024.1335017)
Supplement: Supplementary file 2 [file Table_1.DOCX]

| **Sample** | **Seq_num** | **Base_num** | **Mean_length** |
| --- | --- | --- | --- |
| A1 | 48046 | 19632721 | 408.623 |
| A2 | 37666 | 15361491 | 407.834 |
| A3 | 40305 | 16447481 | 408.075 |
| A4 | 45520 | 18700690 | 410.824 |
| A5 | 41174 | 16773726 | 407.386 |
| A6 | 36364 | 14715538 | 404.673 |
| A7 | 36110 | 14631327 | 405.188 |
| A8 | 34693 | 14091466 | 406.176 |
| A9 | 43450 | 17900911 | 411.989 |
| A10 | 41249 | 16962066 | 411.212 |
| B1 | 39318 | 16250336 | 413.305 |
| B2 | 41926 | 17606898 | 419.952 |
| B3 | 36392 | 15364682 | 422.199 |
| B4 | 39703 | 16758897 | 422.107 |
| B5 | 55702 | 23659481 | 424.751 |
| B6 | 35324 | 14363522 | 406.622 |
| B7 | 38287 | 15546080 | 406.041 |
| B8 | 41669 | 17168211 | 412.014 |
| B9 | 42151 | 17286163 | 410.101 |
| B10 | 47833 | 19784997 | 413.627 |
| C1 | 50148 | 21334020 | 425.421 |
| C2 | 68695 | 29253204 | 425.842 |
| C3 | 44404 | 18853976 | 424.601 |
| C4 | 51512 | 21627458 | 419.853 |
| C5 | 34108 | 14391919 | 421.951 |
| C6 | 35365 | 14906759 | 421.512 |
| C7 | 37322 | 15611993 | 418.305 |

Table S1 The basic information of samples.
